# Stakeholder collaboration in digital climate-health platforms: Mapping patterns across international case studies

**DOI:** 10.1016/j.joclim.2026.100744

**Published:** 2026-09-10

**Authors:** Razieh Rezabeigisani, Sören Becker

**Affiliations:** Department of Geography, Marburg University, Deutschhausstraße 10, 35032 Marburg, Germany

**Keywords:** Digital climate-health platforms, Stakeholder collaboration, Co-design, Digital public health, Qualitative typology, Platform governance

## Abstract

**Introduction:**

Digital climate-health platforms are web, mobile or dashboard-based architectures that link climate, environmental, health and socio-economic data to make climate-related health risks visible and actionable. This study maps stakeholder collaboration patterns in international platforms, focusing on who contributes, how collaboration is organized, and how platform-specific features shape inclusion, decision-making and use across varied institutional contexts.

**Methods:**

We employed a sequential mixed-method design combining internet-based scoping, survey responses and 22 semi-structured interviews across 20 projects. Survey data were analyzed descriptively, and interview material was coded inductively to construct ideal-type collaboration patterns. Findings were triangulated with project documents and platform information to identify stakeholder roles, collaboration mechanisms and case-level pattern assignments.

**Results:**

Digital climate-health platforms involved developers, governments, international agencies, researchers, industry partners, community organizations and end-users. Five ideal-types were identified: central coordination, hierarchical distribution, network-based distribution, iterative co-evolution, and co-leadership. These patterns were not mutually exclusive; each case was assigned to a dominant pattern while secondary features were recorded. Collaboration was shaped by authority over data access, indicators, models, interfaces, feedback interpretation, implementation and post-launch maintenance.

**Conclusion:**

The findings show that collaboration in digital climate-health platforms is both a platform design and a governance issue. Centralized and hierarchical arrangements provide accountability, continuity and authoritative communication but limit end-user influence. Network-based, iterative, and co-leadership arrangements can widen participation and responsiveness, yet they do not automatically constitute co-production. The findings point to the importance of clarifying decision rights, embedding co-design, and evaluating who can influence data, models, interfaces and use.

## 1 Introduction

Digital technologies are increasingly adopted as part of climate change adaptation strategies in healthcare systems and vulnerable communities [[1], [2], [3], [4], [5]]. They support public health functions including epidemiological surveillance, exposure monitoring, contact tracing, case identification, immunization services, and public communication [6,7]. We define digital climate health platforms as web-based, mobile or dashboard-based digital architectures that collect, link, process and communicate climate and environmental data, and, where relevant, combine them with health and socio-economic data to support analysis, decision-making, and policy responses to climate-related health risks. These platforms translate climate-sensitive hazards, such as heat, air pollution, flooding, vector-borne disease or harmful algal blooms, into information for authorities, practitioners, communities or affected individuals.

This definition locates climate-health platforms within research debates on digital public health and platform studies. Digital public health promotes digital technologies for population-based prevention, surveillance and health promotion [8], while digital health also includes clinical infrastructures such as electronic medical records and clinical decision-support systems [9]. Our focus is narrower; we examine platforms whose primary purpose is to make climate-related health risks visible, actionable and governable across institutional settings. Platform studies are relevant because platforms are not just technical websites or applications; they are programmable architectures that organize interactions between heterogeneous actors, and structure data flows through interfaces, standards, algorithms and visualizations. Importantly, they also depend on governance arrangements concerning access, ownership, interoperability, responsibility and trust [[10], [11], [12]].

We conceptualize the development of climate–health platforms as a collaboration process. Collaboration is used as the broad term for sharing knowledge, skills, resources or responsibilities among actors. Research debates stress different levels of involvement in such processes. In digital interface development, co-design refers to a specific design process through which users, designers and domain experts jointly define problems, articulate requirements, develop prototypes and shape implementation pathways [13,14]. According to the WHO, co-design extends beyond feedback by linking needs assessment, stakeholder engagement, implementation and continuous improvement [15]. Co-production is used more cautiously for cases in which actors jointly define problems, integrate knowledge and share responsibility for producing context-specific knowledge, services or decisions [[14], [15], [16], [17]]. This distinction matters because literature shows that user-centered design, usability testing, feedback, trust, data integration and attention to digital divides are central to effective digital health tools and warning systems [8,13,18,19].

Despite growing research on digital climate-health technologies, limited systematic evidence exists on who contributes to their development, how collaboration is organized, and how platform-specific features shape inclusion and use [18,20]. To address this gap, we analyzed 20 international digital climate-health platforms identified through best-practice research. We pursued the following research questions:

− Who are the key stakeholders and which roles do they adopt in digital climate-health platforms?
− What collaboration patterns can be identified in these projects?
− Which patterns are associated with stakeholder influence and effective use?

## 2 Methods

For assessing actor constellations and collaboration patterns in digital climate-health platforms, we employed a sequential, mixed-methods design combining scoping research, survey data and interviews [[21], [22], [23]]. First, we conducted internet-based scoping research using Google and project databases from WHO, the European Environment Agency, and the European Commission to identify digital climate-health platforms across North America, Europe, Australia, and New Zealand. Search terms combined climate-health keywords, such as “climate health”, “heat health”, “air quality health”, “wildfire smoke health”, “climate adaptation health”, and “environmental health”, with digital-tool terms, such as “platform”, “dashboard”, “app”, “portal”, “early warning system”, “decision support tool”, and “risk map”.

We retained projects that were publicly identifiable, digital, concerned with climate-related health risks and oriented to information exchange, warning, mapping or decision support. We excluded clinical systems, static reports and tools without an explicit climate-health function. Telemedicine, patient monitoring, electronic medical records, patient administration systems and clinical decision-support tools were treated as wider digital health infrastructures and included when they explicitly connected climate-health data, actors or decision contexts. This process identified 105 platforms, coded by country, technology type, function, target users and organizational information. The scoping relied mainly on English-language and public material, though initial searches in French and Spanish were conducted too; therefore, non-English initiatives may be underrepresented.

Second, we distributed an online survey to personnel associated with all identified projects between 1 December 2024 and 7 February 2025. The survey collected information on the project’s functions, main stakeholders, data practices, user groups and collaboration. A response was considered valid when all questions were completed and when the response could be linked to a unique project. In total, 28 valid responses were received, yielding a response rate of 26.7%. The survey was used as a mapping and sampling instrument rather than as a representative dataset. The response rate is a limitation, as more visible projects may have been more likely to respond.

Third, 20 projects were selected for interviews through purposeful sampling, informed by survey responses and chosen to maximize variation in geography, platform type, stakeholder constellation, and forms of involvement. 22 semi-structured interviews were conducted between April and September 2025 with project managers, coordinators, data analysts, and researchers. Sixteen interviews were conducted individually, three as group interviews, and three with the leadership team of one project. Interviews were recorded with informed consent and transcribed in clean verbatim form.

The qualitative materials were analyzed in MAXQDA24, applying an inductive multi-stage coding strategy informed by qualitative content analysis, Gioia-style data-structure logic and ideal-type analysis [[24], [25], [26]]. The first author conducted initial coding. Coding decisions, case summaries and uncertain assignments were discussed among the authors through a reflexive consensus process, with interpretive differences resolved through discussion. This process was used to refine and challenge interpretations rather than to assess formal inter-coder reliability. First-order empirical codes remained close to the material and captured practices such as platform coordination, data control, user feedback, prototype testing, advisory structures and shared problem definition. These codes were clustered into focused categories, and higher-order dimensions, including coordination authority, institutional hierarchy, networked interdependence, design temporality and shared agency.

**Fig. 1:**
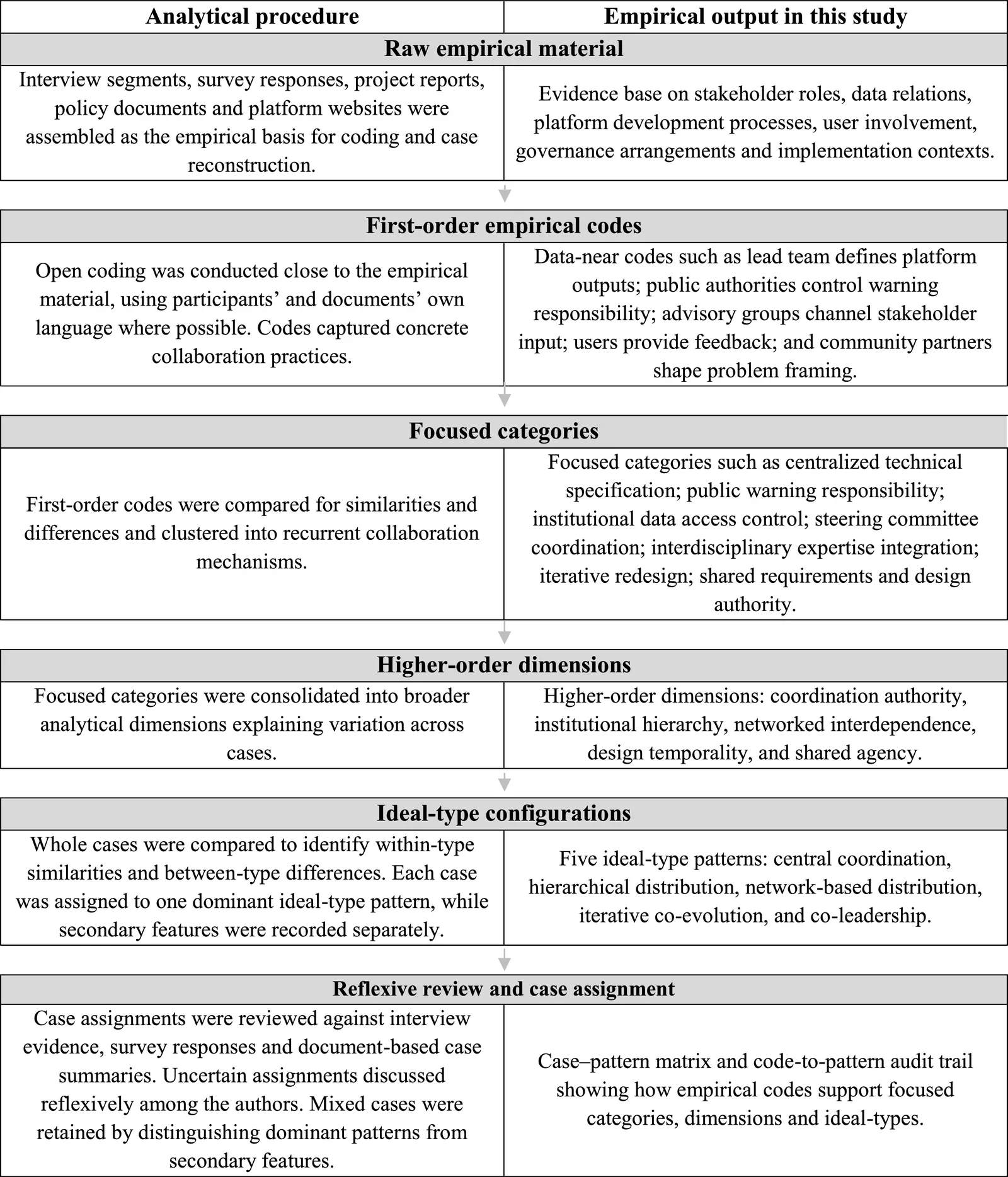
Constructing collaboration ideal-type patterns in digital climate–health platforms, adapted from Gioia-style, data-structure logic and ideal-type analysis [25,26].

**Fig. 2:**
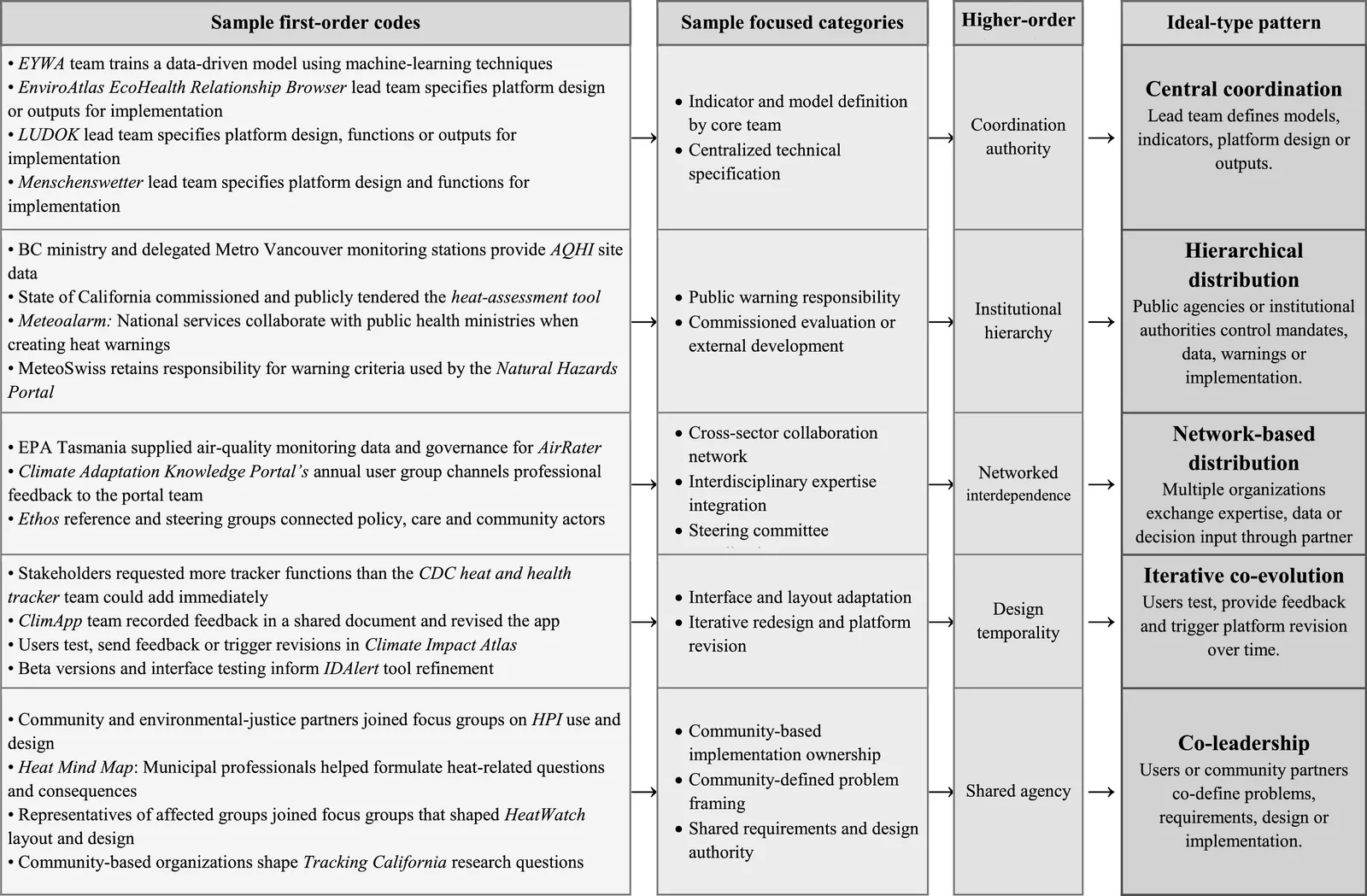
Data structure linking codes, dimensions and ideal-types.

The five collaboration patterns were constructed through cross-case comparison. Cases were not treated as homogeneous or mutually exclusive, as several projects showed mixed features. Each case was assigned to a dominant collaboration pattern according to coordination and decision-making, timing and the degree of stakeholder involvement in development stages, and whether the platform development was linear, iterative or shared. Secondary features were recorded separately. The patterns are ideal-types rather than rigid categories [26]. A pattern was retained only when it demonstrated recurrence across multiple cases, conceptual distinction from the other patterns, and empirical support in the interview, survey and document material. Central coordination was retained because it recurred as an analytically distinct secondary configuration across six cases, despite not being dominant in any case. Fig. 1 presents the workflow for constructing the ideal-type patterns. Fig. 2 shows the data structure linking first-order codes, focused categories, higher-order dimensions and ideal-types.

## 3 Results

### 3.1 Platform ecosystems and stakeholder contributions

**Fig. 3:**
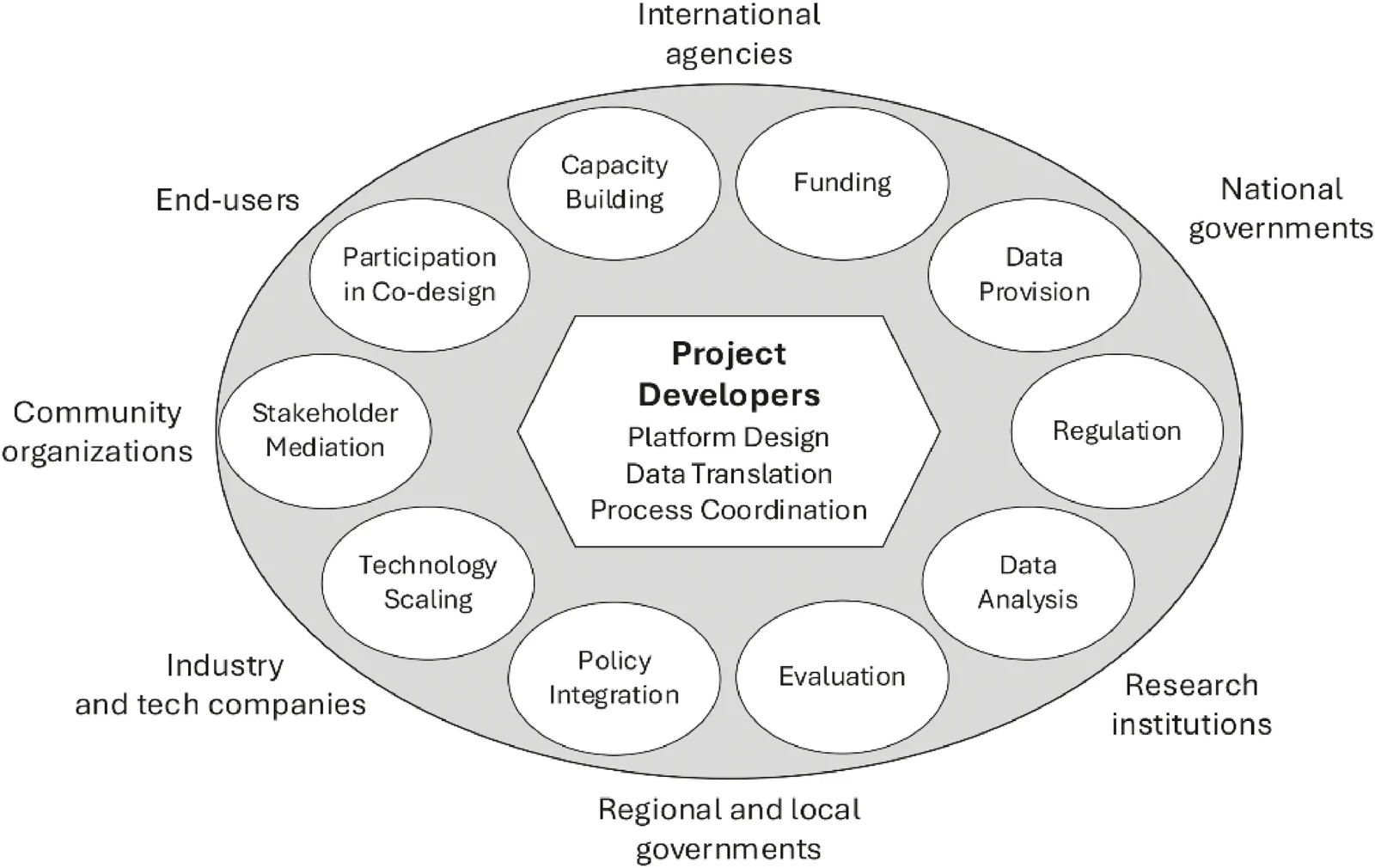
Actor ecosystem of digital climate-health platforms. Different actors may contribute to similar activities, while platform developers often mediate between data, technical infrastructure and user-facing outputs.

The collaboration patterns identified in this study are grounded in the broader actor ecosystems through which digital climate-health platforms are developed, maintained and used. These platforms involve diverse actors, resources and roles across the platform lifecycle [4,20]. Different actor groups contributed to the collection, processing, interpretation and communication of climate, environmental, health and socio-economic data [4]. These contributions included technical development, data access, indicator development, interface design, user communication and implementation [11,27]. Collaboration therefore relied on an "ecosystem" of relationships among actors, some formalized through project mandates, funding arrangements or institutional responsibilities, and others organized through flexible forms of consultation, testing or feedback [10,11,27]. Developers occupied a central and mediating position. They translated heterogeneous datasets into digital interfaces, indicators, maps, and alert protocols, and coordinated interactions between data providers, institutional partners and users. Fig. 3 summarizes the main actor groups in the climate-health platform ecosystem and their contributions.

Actor roles and contributions can be summarized as follows:

• **Platform developers:** These are professionals from informatics, public health, environment, engineering, data science or policy, often affiliated with non-profits, research institutes, or universities. They integrate heterogeneous datasets, translate them into indicators, interfaces or actionable risk outputs, and coordinate stakeholder input through design, testing or feedback processes.
• **International agencies**: WHO, World Meteorologic Organization (WMO), United Nations Developmen Program (UNDP), European Economic Area (EEA), and similar organizations provide data and standards, funding, technical support, and policy frameworks that shape platform scope.
• **National governments and agencies**: Ministries, environmental and health authorities, and meteorological services define strategic and legal frameworks, provide regulatory guidance, commission platform development, and act as data gatekeepers.
• **Regional and local governments:** These implement platforms locally, integrate them into governance structures, interpret outputs for local use, and engage stakeholders through consultations and feedback loops.
• **Researchers:** Researchers design and validate models, interpret data, define indicators, and contribute disciplinary expertise to climate-health interpretation.
• **Industry and technology partners:** Partners provide technical expertise, software infrastructure, support scaling, and cross-sectoral technologies.
• **Community organizations:** These mediate between technical actors and local users, represent context-specific concerns, and support monitoring, communication and feedback.
• **End-users**: End-users participate in testing, workshops, or feedback processes, provide information on usability, accessibility and contexts of use.

### 3.2 Patterns of collaboration

Digital climate-health platforms bring together actors who exchange knowledge and negotiate data access, interface requirements, indicators, models, communication formats and implementation responsibilities [4,14,27]. The collaboration patterns describe how authority and technical decisions are organized around data integration, modeling, visualization, user feedback and maintenance [10,14,19,28]. Projects were assigned to one dominant pattern according to the evidence on who defined platform outputs, controlled data, shaped design decisions and interpreted user input.

"Central coordination" describes platforms in which a lead organization, developer team, or project coordinator manages stakeholder input and retains authority over platform design and functionality. The lead team defines models, indicators, graphic outputs or technical formats through which climate-health risks are visualized. Central coordination is found in processes solving technical issues, while technical solutions can shape collaboration arrangements. In our analysis, central coordination was not the dominant configuration in any individual case, but appeared as a main secondary feature across six cases. Its recurrence reflects a distinct form of concentrated technical authority that can be embedded within otherwise hierarchical, network-based or iterative arrangements. It allows coordination, consistent communication and standardized dataset integration. However, stakeholder involvement often remains consultative. Users, partners or affected groups may provide feedback, but rarely influence analytical choices through which risks are classified or displayed.

"Hierarchical distribution" refers to platforms embedded in public-authority structures. Decision-making is distributed across governance levels, agencies or institutional data providers, but within a top-down arrangement. Public bodies may commission the platform, define warning responsibilities, regulate data access or determine implementation pathways. This pattern provides accountability, continuity and legitimacy when platforms communicate public risks. However, it is constrained by mandates, data-use agreements and approval procedures. Peripheral actors and end-users may be represented indirectly, while authority over defining risk categories remains concentrated at higher governance levels.

"Network-based distribution" describes platforms governed through a network of organizations, including research institutes, public agencies, technology partners, advisory boards, steering committees, or professional communities. Collaboration depends on cross-sectoral interdependence; one actor may provide climate data, another health interpretation, another interface development, and others dissemination or policy relevance. This pattern enables interdisciplinary exchange and problem-solving. Yet network relations remain unequal. Agenda setting often lies with the consortium, while peripheral actors contribute data, expertise or feedback without equivalent influence.

"Iterative co-evolution" captures platforms developed through cycles of design, testing, use and revision. User needs, usability problems, bug reports, accessibility requirements, analytics, workshops or beta testing inform changes. Its contribution lies in enabling platforms to be revised after testing and use, especially for usability, accessibility, communication and interface design. However, feedback does not automatically redistribute authority. Developers decide which feedback becomes technically feasible, scientifically valid or institutionally acceptable. Iteration may improve usability while leaving decisions about data selection, modeling and risk framing unchanged.

"Co-leadership" refers to cases in which users, community organizations or stakeholder representatives participate in defining problems and requirements, design choices or implementation strategies. It is characterized by community-led problem framing and implementation, where local partners shape fieldwork, data measures, interface layout or health-risk interpretation. Co-leadership involves more substantive influence over what the platform is meant to do and for whom. However, institutional hierarchies, unequal technical capacities and resource asymmetries may shape whose knowledge is translated into platform functions.

Table 1 shows collaboration ideal-types and classification criteria.

**Table 1:** Ideal-type collaboration patterns, platform-specific classification evidence and dominant cases.

Ideal-type collaboration pattern	Conceptual Schematic	Key Features	Platform-specific evidence used for classification	Dominant cases
**Central Coordination**		Lead team defines models, indicators, platform design or outputs.	Lead team controls model design, output format, risk indicators, visualization choices or platform architecture; other actors provide data, advice or feedback.	No dominant case; retained as an ideal-type because it recurred as a secondary configuration in six cases.
**Hierarchical Distribution**		Public authorities, agencies, or institutional data holders control mandates, warnings, funding, data access or implementation.	Data access depends on institutional approval; warning authority remains with public agencies; platform scope follows administrative responsibilities or formal mandates.	Air Quality Health Index (AQHI); California Heat Assessment Tool; LUDOK; Meteoalarm; Swiss Natural Hazards Portal
**Network-Based Distribution**		Multiple organizations contribute complementary expertise, data or legitimacy through formal or semi-formal coordination structures.	Advisory boards, steering groups, consortia, APIs, shared databases or professional networks organize data exchange and platform development.	AirRater; Climate Adaptation Knowledge Portal; EYWA; Ethos; Menschenswetter; Predicted Heat Strain app
**Iterative Co-Evolution**		Platform development changes through repeated testing, feedback, usability work or revision cycles.	User analytics, bug reports, beta testing, workshops, surveys or support channels lead to changes in interface design, data presentation or platform functions.	CDC Heat and Health Tracker; California Healthy Places Index; ClimApp; Climate Impact Atlas; EnviroAtlas EcoHealth Relationship Browser; IDAlert
**Co-Leadership**		Users, communities or stakeholder representatives influence problem framing, requirements, design choices or implementation strategy.	Community partners shape data measures, fieldwork, interface layout, risk interpretation, communication priorities or contexts of use before or during platform development.	Heat Mind Map; HeatWatch; Tracking California

Table 2 presents the case-pattern matrix, distinguishing dominant and secondary features.

**Table 2:** Case-pattern matrix of dominant and secondary collaboration ideal-types.

Project case	Central coordination	Hierarchical distribution	Network-based distribution	Iterative co-evolution	Co-leadership
Air Quality Health Index (AQHI)		**Dominant**		Secondary	
AirRater		Secondary	**Dominant**	Secondary	Secondary
CDC Heat and Health Tracker		Secondary	Secondary	**Dominant**	
California Healthy Places Index	Secondary	Secondary	Secondary	**Dominant**	Secondary
California Heat Assessment Tool		**Dominant**		Secondary	
ClimApp				**Dominant**	
Climate Adaptation Knowledge Portal		Secondary	**Dominant**	Secondary	
Climate Impact Atlas		Secondary	Secondary	**Dominant**	
EYWA	Secondary	Secondary	**Dominant**	Secondary	
EnviroAtlas EcoHealth Relationship Browser	Secondary	Secondary	Secondary	**Dominant**	
Ethos		Secondary	**Dominant**	Secondary	
Heat Mind Map		Secondary	Secondary	Secondary	**Dominant**
HeatWatch			Secondary	Secondary	**Dominant**
IDAlert		Secondary	Secondary	**Dominant**	Secondary
LUDOK	Secondary	**Dominant**	Secondary	Secondary	
Menschenswetter	Secondary		**Dominant**	Secondary	
Meteoalarm		**Dominant**	Secondary	Secondary	
Predicted Heat Strain app	Secondary		**Dominant**	Secondary	
Swiss Natural Hazards Portal		**Dominant**	Secondary	Secondary	
Tracking California		Secondary	Secondary	Secondary	**Dominant**

## 4 Discussion

Developing digital platforms for climate-health involves multi-level, cross-sectoral collaboration between actors who hold technical, institutional, scientific and contextual knowledge. The findings highlight that collaboration is not only a question of stakeholder inclusion, but of how authority is distributed across data access, interface design, modeling, feedback interpretation and implementation. The five ideal types should not be read as a sequence from limited to advanced participation; they represent socio-technical arrangements suited to actor constellations and technical targets.

This interpretation connects climate-health platform development to platform studies. Climate-health platforms resemble the programmable architectures and ecosystems that organize interactions, structure data flows and embed governance choices in technical arrangements [[10], [11], [12]]. In climate-health settings, indicators, thresholds, maps, alerts and dashboards translate climate-sensitive hazards into risk knowledge. Participation therefore concerns influence over the components through which risk is selected, visualized and communicated. This supports digital-design and co-production literature showing that engagement can improve relevance, but influence depends on control over requirements, data, design decisions and implementation pathways [13,19,28].

Central coordination appeared where the target is a coherent solution to a bounded technological development problem, such as integrating datasets, translating models into indicators, building an interface or producing standardized outputs. Its significance lies in concentrated technical authority. Lead teams can maintain consistency between data processing, model assumptions, visualization and communication formats when platform development depends on specialist expertise and aligned design requirements [10,13]. The limitation is that stakeholder involvement may remain consultative. Users or partners may improve usability and communication, while having little influence over decisions that define risk categories, indicators or thresholds. Central coordination therefore highlights the difference between feedback and influence over the technical core.

Hierarchical distribution corresponds to actor constellations in which public agencies, ministries, meteorological services, environmental authorities or health institutions hold formal responsibility for data, warnings or implementation. This pattern is relevant when platforms are embedded in administrative systems and require integration into governance processes. Its strength lies in accountability, continuity and authoritative communication, when outputs inform public warnings, emergency response or health-protection decisions. Guidance on heat-health and multi-hazard warning systems similarly emphasizes clear responsibilities, authoritative communication and trusted coordination between meteorological, health and emergency-management actors [[29], [30], [31], [32]]. The limitation is that institutional validity can restrict flexibility. Decisions about data access, warning responsibility or risk classification may be shaped by mandates and approval procedures more than by user input.

Network-based distribution is relevant when platform development depends on the knowledge base of diverse stakeholders. Climate-health platforms often combine climate science, epidemiology, public health, environmental monitoring, software development, policy knowledge and local implementation expertise. Network-based arrangements connect these capacities through consortia, advisory boards, steering groups or shared databases. This pattern resonates with transdisciplinary climate-health collaboration and platform ecosystem literature, where heterogeneous actors contribute complementary resources through shared infrastructures [4,[10], [11], [12],^20^,^27^]. The limitation is that networked collaboration is not necessarily flat. Actors positioned close to data standards, integration layers, modeling decisions or final interfaces can retain more influence than actors involved through consultation, validation or dissemination.

Iterative co-evolution is suitable where the technical target includes adaptation through use. It captures platforms that change through testing, feedback, analytics, workshops, support channels or beta versions. This pattern matters because digital climate-health platforms are often refined after users encounter them in practice. User-centered design and eHealth engagement literature shows that user input can improve usability, adoption and relevance [13,19]. Climate-service literature similarly shows that trials, errors and revisions can make information more usable [33]. The limitation is the boundary of revision. Feedback often changes interface design, accessibility, communication or navigation, while data selection, model assumptions, thresholds and institutional responsibilities remain less open to change. Iterative co-evolution therefore produces responsiveness, but not necessarily shared authority.

Co-leadership corresponds to actor constellations in which community organizations, user representatives or affected groups contribute to problem framing, data measures, design choices, communication priorities or implementation strategies. Rather than reflecting non-expert participation, these cases show contributions grounded in experiential, local or community-based expertise. Co-leadership is relevant where climate-health risks are unevenly distributed and local experience is needed to understand exposure, vulnerability, communication barriers or feasible responses. This pattern moves participation closer to co-design because affected actors can shape what the platform is meant to do and for whom. Sequenced and tactile co-design can make interdisciplinary design decisions more explicit before interfaces are finalized [34,35]. At the same time, co-leadership does not automatically equal co-production. Critical co-production literature shows that participation can coexist with unequal technical capacity, funding power, institutional mandates and infrastructure control [14,16,28].

The typology contributes to climate-health and digital public health debates by specifying how collaboration is organized through platform design. Calls for interdisciplinarity, user-centered design, trust and digital inclusion are often framed as general requirements; this study shows how they are organized in practice [13,[18], [19], [20]]. These requirements take different forms depending on the platform arrangement: technical coherence, administrative validity, knowledge integration, responsiveness, and contextual relevance. For practice and evaluation, the typology shifts attention from stakeholder presence to stakeholder influence. A platform may involve many actors while leaving key decisions concentrated in a lead team or public authority. The typology therefore makes visible how influence is distributed across the platform lifecycle, from defining indicators and validating models to controlling data access, revising interfaces, interpreting feedback and ensuring maintenance.

## 5 Conclusion

This study examined how collaboration is organized in digital climate-health platforms and what this reveals about the relationship between platform design, knowledge production and public health governance. The findings suggest that collaboration patterns prevail depending on actor constellations, technical functions and project goals. Across international cases, we identified five ideal-type patterns: central coordination, hierarchical distribution, network-based distribution, iterative co-evolution and co-leadership. These patterns show that collaboration is not defined simply by stakeholder diversity; it is shaped by authority over data access, indicator selection, modeling, interface design, feedback interpretation and long-term maintenance.

The paper contributes to climate-health, digital public health and platform studies. First, it clarifies the actor ecosystem through which climate, environmental, health and socio-economic data are translated into platform outputs. Second, it offers a typology for comparing collaboration arrangements without treating them as fixed categories. Third, it shows why participation, co-design and co-production should be distinguished in studying digital climate-health platforms. Inclusive platform development requires attention to decision rights, technical translation processes and post-launch adaptation. Future evaluation should examine not only who is consulted, but also who can shape design choices, use, revision and maintenance after launch.

## Authorship statement

The authors have jointly worked in the conception and realization of this paper. The underlying empirical research was jointly supervised by both authors.

## CRediT authorship contribution statement

**Razieh Rezabeigisani:** Writing – review & editing, Writing – original draft, Visualization, Resources, Methodology, Investigation, Formal analysis, Conceptualization. **Sören Becker:** Writing – review & editing, Resources, Methodology, Investigation, Formal analysis, Data curation, Conceptualization.

## Declaration of competing interest

The authors declare no competing interests relevant to the content of this paper.

## Data Availability

The data that support the findings of this study are available from the corresponding author upon reasonable request. Aggregated data and anonymized excerpts used for analysis are provided within the article.
