# Supplementary material for: Stakeholder collaboration in digital climate-health platforms: Mapping patterns across international case studies
Source: J Clim Chang Health. 2026 Sep 10;31:100744. doi: 10.1016/j.joclim.2026.100744 (PMC13583760; doi:10.1016/j.joclim.2026.100744)
Supplement: Supplementary file 1 [file mmc1.docx]

**Supplementary Table 1. Case studies and interview characteristics**

| **No.** | **Project name** | **Country/region** | **Responsible organization(s)** | **Platform type** | **Primary function** | **Interview mode** | **Interviewee role(s)** | **Interview type** |
| --- | --- | --- | --- | --- | --- | --- | --- | --- |
| 1 | Air Quality Health Index (AQHI) | Canada | Environment and Climate Change Canada, with Health Canada; developed by health and environmental professionals and delivered through Government of Canada air-quality services | Early warning system | Air-quality health index; public communication and forecasting tool | Online | Project Manager | Individual |
| 2 | AirRater | Australia | Menzies Institute for Medical Research, University of Tasmania, with CSIRO and government partners | Early warning system | Air-quality, pollen, smoke, heat and symptom app | Online | Project Manager | Individual |
| 3 | California Healthy Places Index | United States | Public Health Alliance of Southern California; fiscally administered by the Public Health Institute | Vulnerability assessment and adaptation tool | Place-based health equity and extreme-heat vulnerability platform | Online | Former Project Manager; State Administration Representative | Group |
| 4 | California Heat Assessment Tool (Cal-Heat) | United States | Funded by the California Natural Resources Agency; developed by Four Twenty-Seven with Argos Analytics, Habitat Seven and Public Health Institute | Vulnerability assessment and adaptation tool | Heat assessment and adaptation tool | Online | Project Manager | Individual |
| 5 | CDC Heat and Health Tracker | United States | U.S. Centers for Disease Control and Prevention, National Environmental Public Health Tracking Program | Vulnerability assessment and adaptation tool | National heat-health dashboard and tracker | Online | Project Manager; Data Lead | Group |
| 6 | ClimApp | Across Europe | ClimApp project consortium; project coordinated by Chuansi Gao at Lund University | Early warning system | Heat-stress and climate adaptation app | Online | Project Manager | Individual |
| 7 | Climate Adaptation Knowledge Portal | Netherlands | Climate Adaptation Services, managed on behalf of the Dutch Ministry of Infrastructure and Water Management | Vulnerability assessment and adaptation tool | Climate adaptation knowledge portal | In person | Communication Specialist and Content Developer | Individual |
| 8 | Climate Impact Atlas | Netherlands | Climate Adaptation Services, commissioned by the Dutch Ministry of Infrastructure and Water Management | Vulnerability assessment and adaptation tool | Climate risk and adaptation map atlas | In person | Data Analyst | Individual |
| 9 | EnviroAtlas and EcoHealth Relationship Browser | United States | U.S. Environmental Protection Agency, EnviroAtlas; Office of Research and Development | Dashboards, databases and browsers | Ecohealth evidence browser and environmental health platform | Online | Senior Environmental Health Scientist | Individual |
| 10 | Ethos Project | Australia | Griffith University; Ethos Project Team | Early warning system | Heat-health early warning and older-adult heat preparedness technology | In person | Project Manager; Project Lead 1; Project Lead 2 | Individual |
| 11 | EYWA | Across Europe | National Observatory of Athens; BEYOND Centre of Earth Observation Research and Satellite Remote Sensing; EuroGEO Action Group “Earth Observation for Epidemics of Vector-borne Diseases” | Early warning system | Mosquito-borne disease early warning system | Online | Senior Expert and Project Contributor | Individual |
| 12 | Heat Mind Map | Netherlands | Amsterdam University of Applied Sciences; Climate Adaptation Services; Municipality of Amsterdam | Vulnerability assessment and adaptation tool | Interactive urban heat consequences and adaptation mind map | In person | Project Manager | Individual |
| 13 | HeatWatch | Australia | University of Sydney; Heat and Health Research Centre; Sydney Environment Institute | Early warning system | Personalised heat-health risk and cooling-recommendation app | In person | Project Manager | Individual |
| 14 | IDAlert | Across Europe | Umeå University; IDAlert consortium | Early warning system | Climate-sensitive infectious disease decision-support and risk-forecasting tool | Online | Project Manager | Individual |
| 15 | LUDOK | Switzerland | Swiss Tropical and Public Health Institute, on behalf of the Swiss Federal Office for the Environment | Dashboards, databases and browsers | Environmental health evidence database and knowledge platform | Online | Project Manager | Individual |
| 16 | Menschenswetter | Germany | memepublisher GmbH, in cooperation with the German Weather Service (DWD) | Dashboards, databases and browsers | Weather-health information website and patient-oriented forecast interpretation | Online | Project Manager | Individual |
| 17 | Meteoalarm | Across Europe | EUMETNET; European National Meteorological and Hydrological Services network; warnings supplied by participating European National Meteorological and Hydrological Services | Early warning system | European multi-hazard warning aggregation platform | Online | Project Manager | Individual |
| 18 | PHS — Predicted Heat Strain app | Australia | University of Queensland, Faculty of Science, and Rio Tinto; developed by Stellar Science LTD | Early warning system | Occupational heat-stress assessment and decision-support app | In person | Project Lead | Individual |
| 19 | Swiss Natural Hazards Portal | Switzerland | Swiss federal natural-hazard authorities, including MeteoSwiss, FOEN, Swiss Seismological Service and WSL Institute for Snow and Avalanche Research SLF | Early warning system | National natural hazards warning portal | Online | Project Manager | Individual |
| 20 | Tracking California | United States | Public Health Institute, in partnership with the California Department of Public Health and CDC’s National Environmental Public Health Tracking Program | Vulnerability assessment and adaptation tool | Environmental public health tracking and community data platform | Online | Policy Director; Climate Data Analyst; Research Data Analyst; Research Scientist | Group |
