# Supplementary material for: Stakeholder collaboration in digital climate-health platforms: Mapping patterns across international case studies
Source: J Clim Chang Health. 2026 Sep 10;31:100744. doi: 10.1016/j.joclim.2026.100744 (PMC13583760; doi:10.1016/j.joclim.2026.100744)
Supplement: Supplementary file 2 [file mmc2.docx]

**Supplementary Table 2. Case vignettes and evidence used for pattern assignment**

| **Project (case)** | **Dominant pattern** | **Secondary pattern(s)** | **Confidence** | **Case vignette** | **Evidence sources** |
| --- | --- | --- | --- | --- | --- |
| AQHI | Hierarchical distribution | Iterative co-evolution | Medium | AQHI illustrates a hierarchical distribution pattern because the production and circulation of risk information are anchored in public air-quality and health authorities. The evidence links AQHI to ministry and provincial structures, delegated monitoring stations and Health Canada’s role in developing the health evidence behind the index. Collaboration is therefore not absent, but it is organized through a public mandate and an authoritative warning infrastructure. The case also shows an iterative layer: regular meetings, digital communication channels, website analytics and mailbox questions during wildfire season informed how the service was adjusted. | 4 interview segments; 1 survey response. Coded indicators: Public warning responsibility; Iterative redesign and platform revision; Analytics and support-channel learning. Pattern signal: Hierarchical distribution (3); Iterative co-evolution (2). |
| AirRater | Network-based distribution | Hierarchical distribution; Iterative co-evolution; Co-leadership | Medium | AirRater is best read as a network-based platform because its development depended on recurring relations among government agencies, academic actors, data providers, health departments, technology developers and users. Funding and commissioning shaped the app’s expansion beyond Tasmania, but the platform’s practical functioning relied on partners supplying monitoring data, health expertise, promotion, feedback and implementation support. The evidence also indicates repeated bug fixing and upgrades based on user feedback. A weaker co-leadership trace appears in the advisory role of early users and board members, but this does not dominate the case. | 16 interview segments; 3 survey responses. Coded indicators: Iterative redesign and platform revision; Cross-sector partner interdependence; Commissioned evaluation or external development; Grant-funded platform development; Cross-sector collaboration network. Pattern signal: Network-based distribution (8); Hierarchical distribution (6); Iterative co-evolution (4); Co-leadership (1). |
| CDC Heat and Health Tracker | Iterative co-evolution | Hierarchical distribution; Network-based distribution | High | The CDC Heat and Health Tracker is a strong example of iterative co-evolution. The platform was launched through CDC programs and uses authoritative heat-risk and institutional data sources, but the dominant empirical signal is repeated adjustment around user needs. Stakeholders requested additional functions, media use during heat season created new communication demands and internal review processes were used when external user research required additional approvals. The case combines climate science, public health, engineering, data science, urban planning and social-science expertise, so networked expertise matters. However, what distinguishes the case is not only the diversity of actors but the way the interface, functions and communication practices were revised through use, feedback and changing seasonal demand. | 16 interview segments; 3 survey responses. Coded indicators: Iterative redesign and platform revision; Interface and layout adaptation; Interdisciplinary expertise integration; Cross-sector partner interdependence; Analytics and support-channel learning. Pattern signal: Iterative co-evolution (11); Network-based distribution (5); Hierarchical distribution (3). |
| California Healthy Places Index | Iterative co-evolution | Central coordination; Hierarchical distribution; Network-based distribution; Co-leadership | Medium | The California Healthy Places Index combines a centrally defined indicator architecture with extensive iterative adaptation. The initial framing drew on literature and social-determinants reasoning, while state funding and university contracts shaped the development of related climate tools. Local health-department steering structures, community organizations and environmental-justice partners contributed to focus groups, indicator discussions, messaging and use. The case therefore contains traces of central coordination, hierarchy, networked expertise and shared agency. It is assigned to iterative co-evolution because the evidence repeatedly points to versioning, user testing, feedback forms, training activities and design revision. | 19 interview segments; 3 survey responses; 2 website/document/report content-analysis items. Coded indicators: Iterative redesign and platform revision; Commissioned evaluation or external development; Steering committee coordination; Grant-funded platform development; Interface and layout adaptation. Pattern signal: Iterative co-evolution (8); Hierarchical distribution (7); Network-based distribution (7); Central coordination (1); Co-leadership (1). |
| California Heat Assessment Tool (Cal-Heat) | Hierarchical distribution | Iterative co-evolution | Low | The California Heat Assessment Tool is presented cautiously because the workbook contains limited coded evidence for this case. The available material points to a hierarchical distribution pattern: the tool was commissioned by the State of California through a public tendering process and developed through external technical expertise. A secondary iterative element appears because stakeholder needs were assessed before the tool was realized. This is enough to justify a provisional vignette about commissioned platform development, but not enough to infer a broader collaboration ecology or sustained user-feedback loop. | 6 interview segments. Coded indicators: State-commissioned tool development; Public tendering process; External technical development; Public-agency mandate and scope setting; Stakeholder needs assessment before realization; Interface and layout adaptation. Pattern signal: Hierarchical distribution (5); Iterative co-evolution (1). |
| ClimApp | Iterative co-evolution | No secondary pattern | Low | ClimApp is a limited-evidence case in the coded material. The retained evidence indicates that the team recorded feedback in a shared document and revised the app accordingly. This supports a cautious assignment to iterative co-evolution because feedback was documented, reviewed and linked to subsequent modification. However, the evidence does not support stronger claims about hierarchy, network governance, central coordination or co-leadership. | 5 interview segments. Coded indicators: User feedback recorded in a shared document; Feedback review by the development team; App revision after feedback; Iterative redesign and platform revision; Feedback-linked modification. Pattern signal: Iterative co-evolution (5). |
| Climate Adaptation Knowledge Portal | Network-based distribution | Hierarchical distribution; Iterative co-evolution | Medium | The Climate Adaptation Knowledge Portal represents network-based distribution because it connects public commissioning, professional user groups and multiple knowledge communities around adaptation information. The Dutch ministry or Rijkswaterstaat frames and commissions the portal, which gives the case a hierarchical layer. Yet the dominant pattern lies in advisory or steering structures, annual user groups and the connection of municipalities, water boards and knowledge institutions. Feedback is also gathered through surveys and communication channels, producing an iterative layer. | 18 interview segments. Coded indicators: Interdisciplinary expertise integration; Interface and layout adaptation; Commissioned evaluation or external development; Steering committee coordination; Iterative redesign and platform revision. Pattern signal: Network-based distribution (7); Iterative co-evolution (7); Hierarchical distribution (4). |
| Climate Impact Atlas | Iterative co-evolution | Hierarchical distribution; Network-based distribution | High | The Climate Impact Atlas is a high-confidence example of iterative co-evolution. The case includes institutional data-control arrangements and combines climate science, public health, engineering, social science and policy expertise. Government agencies, academic institutions and end users appear as relevant stakeholder groups. Still, the strongest evidence concerns user needs, interface adaptation, regular meetings, surveys, training and feedback-based revision. The platform is therefore not only a repository of climate-impact information but a changing interface shaped by professional and end-user engagement. | 6 interview segments; 3 survey responses; 1 website/document/report content-analysis item. Coded indicators: Interface and layout adaptation; Analytics and support-channel learning; Iterative redesign and platform revision; Data-use agreement and access control; Climate-health expertise integration. Pattern signal: Iterative co-evolution (6); Network-based distribution (3); Hierarchical distribution (1). |
| EYWA | Network-based distribution | Central coordination; Hierarchical distribution; Iterative co-evolution | Medium | EYWA is a network-based case built around international Earth-observation and public-health collaboration. A core team trains a data-driven model using machine-learning techniques, and institutional data holders regulate which epidemiological data enter the modeling process. These features introduce central coordination and hierarchy. However, the platform depends on climate-health expertise, public-health authorities, academic partners, local communities and health experts who test and refine the tool. Feedback is used to assess accuracy and improve the interface. | 20 interview segments; 3 survey responses; 2 website/document/report content-analysis items. Coded indicators: Interdisciplinary expertise integration; Interface and layout adaptation; Data-use agreement and access control; Indicator and model definition by core team; Commissioned evaluation or external development. Pattern signal: Network-based distribution (9); Iterative co-evolution (8); Hierarchical distribution (6); Central coordination (2). |
| EnviroAtlas EcoHealth Relationship Browser | Iterative co-evolution | Central coordination; Hierarchical distribution; Network-based distribution | High | The EnviroAtlas EcoHealth Relationship Browser is a high-confidence case of iterative co-evolution. The platform has a clear federal and technical center: a lead team specifies platform design, and an EPA federal team maintains the browser, literature review and IT hosting. Advisory structures, federal agencies, NGOs, universities and end users add a networked layer. The decisive evidence, however, concerns beta testers, workshops, classroom users and other user-feedback channels that informed changes to the browser. The case therefore shows how a centrally maintained public platform can evolve through practical use and staged feedback rather than through single-stage consultation. | 21 interview segments; 2 survey responses. Coded indicators: Interdisciplinary expertise integration; Iterative redesign and platform revision; Interface and layout adaptation; Analytics and support-channel learning; Centralized technical specification. Pattern signal: Iterative co-evolution (11); Network-based distribution (8); Central coordination (2); Hierarchical distribution (2). |
| Ethos | Network-based distribution | Hierarchical distribution; Iterative co-evolution | Medium | Ethos sits close to the boundary between network-based distribution and iterative co-evolution. The case contains funding and public-authority elements, but its collaboration pattern is dominated by reference and steering groups that connected policy, care-system, technology, community and heat-preparedness actors. The intervention was also refined through surveys, field trials, workshops and work cafés, which explains the strong iterative secondary pattern. The final assignment to network-based distribution is justified because the actor configuration itself appears to organize the work: the intervention is produced through connections among institutions and professional communities, not only through repeated redesign cycles. | 22 interview segments. Coded indicators: Interface and layout adaptation; Steering committee coordination; Iterative redesign and platform revision; Interdisciplinary expertise integration; Cross-sector partner interdependence. Pattern signal: Network-based distribution (10); Iterative co-evolution (10); Hierarchical distribution (2). |
| Heat Mind Map | Co-leadership | Hierarchical distribution; Network-based distribution; Iterative co-evolution | High | Heat Mind Map is the clearest co-leadership case in the workbook. Municipal professionals and community partners did not merely comment on a finished platform; they helped formulate heat-related questions, consequences, problem definitions and implementation choices. Institutional data holders and public authorities still shaped access and mandate, and different professional fields contributed complementary expertise. Yet the dominant empirical signal is shared agency in framing the problem and shaping the platform’s practical orientation. The case distinguishes co-leadership from ordinary user feedback because stakeholders helped define what the platform was for, not only whether the interface was usable. | 15 interview segments. Coded indicators: Community-defined problem framing; Interdisciplinary expertise integration; Shared requirements and design authority; Public warning responsibility; Data-use agreement and access control. Pattern signal: Co-leadership (9); Network-based distribution (3); Hierarchical distribution (2); Iterative co-evolution (1). |
| HeatWatch | Co-leadership | Network-based distribution; Iterative co-evolution | Medium | HeatWatch is assigned to co-leadership because representatives of affected groups, Western Sydney NGOs and grassroots organizations shaped engagement, layout and design during development. The evidence points to focus groups and community testing that generated feedback and affected the tool’s requirements. A networked layer is present because NGOs and grassroots organizations helped build the engagement infrastructure. An iterative layer is also present because testing led to revision. The community role extended beyond consultation, but the available evidence is less extensive than in Heat Mind Map, so the claims should remain moderately framed. | 7 interview segments. Coded indicators: Shared requirements and design authority; Iterative redesign and platform revision; Cross-sector partner interdependence; Community-based implementation ownership. Pattern signal: Co-leadership (4); Iterative co-evolution (2); Network-based distribution (1). |
| IDAlert | Iterative co-evolution | Hierarchical distribution; Network-based distribution; Co-leadership | Medium | IDAlert is an iterative co-evolution case situated within a European institutional and scientific network. Mandated agencies structure the development of the risk tools, and the work combines climate science, public health and data-science expertise. Government agencies and academic institutions appear as key stakeholders. The dominant evidence concerns beta versions, interface testing, workshops and usability requirements that informed refinement. A weak co-leadership trace appears where users or partners influence requirements, but the case is better interpreted as iterative because feedback primarily supports tool refinement rather than shared control over the platform’s problem framing or institutional mandate. | 8 interview segments; 3 survey responses. Coded indicators: Interface and layout adaptation; Interdisciplinary expertise integration; Public warning responsibility; Climate-health expertise integration; Cross-sector partner interdependence. Pattern signal: Iterative co-evolution (5); Network-based distribution (4); Hierarchical distribution (1); Co-leadership (1). |
| LUDOK | Hierarchical distribution | Central coordination; Network-based distribution; Iterative co-evolution | Medium | LUDOK illustrates hierarchical distribution because the platform operates on behalf of the Swiss Federal Office for the Environment and is shaped by public mandate, institutional data access and commissioning arrangements. A core lead team specifies platform design and outputs, while advisory structures, medical and public-health expertise, technology developers and academic actors create a networked support layer. The case also includes accessibility and user-feedback efforts, including surveys and design adaptation. The dominant pattern remains hierarchical because authority, mandate and data governance organize the platform’s operation. | 18 interview segments; 3 survey responses; 1 website/document/report content-analysis item. Coded indicators: Commissioned evaluation or external development; Accessibility and translation adaptation; Interface and layout adaptation; Public warning responsibility; Data-use agreement and access control. Pattern signal: Hierarchical distribution (8); Iterative co-evolution (7); Network-based distribution (6); Central coordination (1). |
| Menschenswetter | Network-based distribution | Central coordination; Iterative co-evolution | Medium | Menschenswetter is a network-based case because it combines meteorological data, medical interpretation and climate-health expertise. The lead team specifies platform design and outputs, which gives the case a central-coordination component. However, the platform’s value is produced through the relation between weather services, medical and public-health knowledge, data-science expertise, physicians and end users. User needs also shape design decisions, and regular meetings or digital communication channels support engagement. | 6 interview segments; 3 survey responses. Coded indicators: Medical and public-health expertise; Iterative redesign and platform revision; Centralized technical specification; Interdisciplinary expertise integration; Climate-health expertise integration. Pattern signal: Network-based distribution (5); Iterative co-evolution (3); Central coordination (1). |
| Meteoalarm | Hierarchical distribution | Network-based distribution; Iterative co-evolution | Medium; interpretive override | Meteoalarm is an important interpretive-override case. A simple frequency count gave more support to iterative co-evolution, but the final assignment to hierarchical distribution is analytically justified because national meteorological services provide warnings and retain responsibility for warning criteria while Meteoalarm aggregates and redistributes them. The platform also depends on a European cross-border warning network, redistributors, ERCC use and APIs. Feedback surveys and interface improvements exist, but the evidence does not indicate a continuous user loop that would displace the public warning mandate. | 12 interview segments; 2 survey responses; 1 website/document/report content-analysis item. Coded indicators: Public warning responsibility; Interface and layout adaptation; Iterative redesign and platform revision; Commissioned evaluation or external development; Steering committee coordination. Pattern signal: Iterative co-evolution (6); Hierarchical distribution (5); Network-based distribution (4). |
| Predicted Heat Strain app | Network-based distribution | Central coordination; Iterative co-evolution | Medium | The Predicted Heat Strain app combines central coordination with network-based distribution. A core team translated ISO 7933 equations into app requirements while a subcontractor built the tool. This gives the platform a strong technical-specification center. At the same time, multiple organizations exchanged data, expertise and decisions, and interface choices were adapted to user needs. The final assignment to network-based distribution is appropriate because the app’s development depended on distributed expertise across occupational-health, technical and implementation actors. | 7 interview segments. Coded indicators: Interdisciplinary expertise integration; Interface and layout adaptation; Centralized technical specification. Pattern signal: Network-based distribution (4); Iterative co-evolution (2); Central coordination (1). |
| Swiss Natural Hazards Portal | Hierarchical distribution | Network-based distribution; Iterative co-evolution | Medium | The Swiss Natural Hazards Portal is a hierarchical distribution case with strong networked and iterative components. MeteoSwiss retains responsibility for warning criteria, and public authority defines the mandate. At the same time, steering structures coordinate specialist departments, and different professional fields contribute expertise. The portal was revised around user expectations through a UX agency, surveys and communication channels. Accessibility requirements also led to sign-language and plain-language content. The dominant pattern remains hierarchical because warning responsibility and mandate organize the platform. | 22 interview segments; 2 website/document/report content-analysis items; 2 survey responses. Coded indicators: Public warning responsibility; Interface and layout adaptation; Interdisciplinary expertise integration; Cross-sector partner interdependence; Steering committee coordination. Pattern signal: Hierarchical distribution (9); Network-based distribution (9); Iterative co-evolution (8). |
| Tracking California | Co-leadership | Hierarchical distribution; Network-based distribution; Iterative co-evolution | Medium; interpretive override | Tracking California is a co-leadership case despite frequency counts that also supported hierarchy and network-based distribution. Funding opportunities and institutional data agreements shape which ideas move forward, and data stewards or advisory groups guide topics and priorities. However, the decisive evidence concerns community-based organizations and public agencies working together, with an Imperial community-based organization leading and supporting local monitoring work. Community partners shaped research questions, problem definitions, design choices and indicator decisions. The assignment therefore rests on the quality of stakeholder agency rather than on the frequency of coded signals alone. | 20 interview segments; 2 website/document/report content-analysis items. Coded indicators: Grant-funded platform development; Cross-sector partner interdependence; Community-based implementation ownership; Data-use agreement and access control; Advisory board mediation. Pattern signal: Hierarchical distribution (7); Network-based distribution (7); Co-leadership (5); Iterative co-evolution (3). |
